# Supplementary material for: Predicting multiple observations in complex systems through low-dimensional embeddings
Source: Nat Commun. 2024 Mar 12;15:2242. doi: 10.1038/s41467-024-46598-w (PMC10933326; doi:10.1038/s41467-024-46598-w)
Supplement: Supplementary file 1 — Supplementary Information [file 41467_2024_46598_MOESM1_ESM.pdf]

Supplementary Information for *Predicting Multiple Observations in Complex Systems through Low-Dimensional Embeddings*

Tao Wu[1], Xiangyun Gao[2,3,*], Feng An[4,*], Xiaotian Sun[2], Haizhong An[2,3], Zhen Su[5,6], Shraddha Gupta[5,7], Jianxi Gao[8,9,*], Jürgen Kurths[5,7,*]

1 College of Management Science, Chengdu University of Technology, Chengdu 610059, China

2 School of Economics and Management, China University of Geosciences, Beijing 100083, China

3 Key Laboratory of Carrying Capacity Assessment for Resource and Environment, Ministry of Land and Resources, Beijing 100083, China

4 School of Economics and Management, Beijing University of Chemical Technology, Beijing 100029, China

5 Potsdam Institute for Climate Impact Research (PIK)–Member of the Leibniz Association, Potsdam 14473, Germany

6 Department of Computer Science, Humboldt University at Berlin, Berlin 12489, Germany

7 Department of Physics, Humboldt University at Berlin, Berlin 12489, Germany

8 Department of Computer Science, Rensselaer Polytechnic Institute, Troy, NY 12180, USA

9 Network Science and Technology Center, Rensselaer Polytechnic Institute, Troy, NY 12180, USA

***E-mail: gxy5669777@126.com, af15910602135@126.com, gaoj8@rpi.edu, kurths@pik-potsdam.de**

1 Supplemental Notes

1.1 Main mathematical symbols in this work

Symbols	Notes

N	The dimension of the original system
L	The length of the time series
E	The embedding dimension
τ	The time lag
$X(t)$ or $Y(t)$	The spatial point
$x(t)$ or $y(t)$	The temporal series
M	The original manifold
M_x	The low-dimensional representation via delay embedding
M_0	The low-dimensional representation via feature embedding
ϕ	A smooth mapping between the original system to its feature manifold
φ	A mapping between the original system and the reconstructed manifold
ψ	A smooth mapping between the feature manifold and reconstructed manifold

$\hat{\psi}$	A mapping from the feature manifold to the final coordinate of the reconstructed manifold
k	The randomly selected k-th data
$RMSE$	The normalized root mean square error (Normalized by the standard deviation of the input series)
ρ	The Pearson correlation between predicted values and observed values
σ	The strength of white noise
η	The proportion of the training sample

1.2 Dataset Details

1.2.1 The 3-dimensional Rössler system

The 3-dimensional Rössler model is selected as a benchmark example to illustrate the mechanism of the FRMM framework. For simplicity, we set the initial values as $(x(0), y(0), z(0)) = (4,4,4)$, respectively, and the integral range is from 10 to 40. Thus, the length of the output time series for each variable is 3489. The embedding dimension is $E = 2$, and the LTSA algorithm is applied here to determine the feature manifold.

$$\begin{cases} \dot{x} = -y - z \\ \dot{y} = x + 0.2y \\ \dot{z} = 0.2 + xz - 5.7z \end{cases} \tag{S1}$$

1.2.2 The wind speed of the Indian Monsoon

For the dataset from the climate system, we use the lower-level (850 hPa) zonal wind component from region IMI2 (70E-90E, 20 N-30 N), with a daily temporal resolution from 1998 to 2019, provided on a spatial grid with a resolution of $1^0 \times 1^0$, from ERA5 Reanalysis data. Then, we generate a 231-dimensional subsystem, where each daily observation has a length of 1094. All the datasets are free at https://cds.climate.copernicus.eu/. We further verify the prediction at IMI2 at a monthly temporal resolution, and each observation has a length of 264. The embedding dimension and time lag are $E = 5, \tau = 5$, respectively, and LLE is used to find their low-dimensional feature manifold.

1.2.3 EEG signal dataset

The 64-dimensional EEG signal series is used to validate our framework in neuroscience. Biosemi Active Two was used to record 64 channels of EEG from thirteen healthy participants (8 females; mean age 22.6; SD 1.04). There are 64 time series for each participant. Each series has a length of 800. These series are typically non-stationary, we normalize the raw data by *Z*-Score normalization. All the datasets are free on the website http://bnci-horizon-2020.eu/database/data-sets. The embedding dimension and time lag are $E = 5, \tau = 5$, respectively, and LTSA is applied here to find the feature manifold.

1.2.4 Exchange rate dataset

Foreign exchange markets constitute a typical complex system, and we select the daily closing price of 70 currencies against the US dollar (USD) as our sample (see Table S1). All the data are free from http://fx.sauder.ubc.ca/data.html. The period is from 20.06.2016 to 29.06.2023. Each observation has a length of 1256. The embedded dimension and time lag are $E = 5, \tau = 5$, respectively, and the ISOMAP algorithm is used to find their low-dimensional feature manifold.

1.2.5 Stock index dataset

In the stock market, we choose a sample of 46 MSCI (Morgan Stanley Capital International) stock indices (Table S2) Their daily closing prices from 2015-01-01 to 2018-11-16 are selected as the sample, where each observation has a length of 1000. All the data are from the Wind database. Here, we select $E = 5, \tau = 5$, respectively, and the Laplacian algorithm is applied to find their low-dimensional feature manifold.

1.2.6 Traffic speed dataset

The traffic speed (mile/h) dataset is collected from 207 loop detectors in the 134-highway of Los Angeles County from 2012-03-01 to 2012-06-30, and each time series has a length of 488. It constitutes a 207-dimensional subsystem. In this work, we use the Laplacian algorithm to embed the original system into a 10-dimensional space.

1.3 Gaussian process regression

As the architecture of the FRMM, finding the mapping between the low-dimensional feature manifold and the reconstructed manifold allows for achieving ahead predictions in high-dimensional systems. Particularly, we need to identify the following mapping:

$$\widehat{\psi}\begin{pmatrix} y_1(1) & y_2(1) & \cdots & y_E(1) \\ \vdots & \vdots & \cdots & \vdots \\ y_1(h) & y_2(h) & \cdots & y_E(h) \\ \vdots & \vdots & \cdots & \vdots \\ y_1(L) & y_2(L) & \cdots & y_E(L) \end{pmatrix} = \begin{pmatrix} x_j(1+(E-1)\tau) \\ \vdots \\ x_j(L) \\ \vdots \\ x_j(L+(E-1)\tau) \end{pmatrix} \qquad \text{(S2)}$$

In other words, our goal is to identify a mapping

$$y_i = f(x_i) + \xi_i \qquad \text{(S3)}$$

where $x_i \in R^E$ and $y_i \in R$. We aim to find a well-fitted mapping *f*. However, it is difficult to find an optimal function with traditional regressions (e.g., least squares regression) in real-world complex systems. Instead, Gaussian process regression contributes to examining the distribution of the mapping f. This approach deals with the mapping as a Gaussian process

$$f(x) \sim GP(u(x), \Sigma(x)),\ Y = f(X) + \varepsilon \sim N(u(x), \Sigma(x) + \sigma^2 I) \tag{S4}$$

For a new sample $x_* = [x_1^*, \cdots, x_i^*, \cdots, x_n^*]'$, our goal is to predict y_* ($y_* = f(x_*) + \varepsilon$). We note that the mapping f is a Gaussian process, which denotes a joint distribution

$$\begin{bmatrix} Y \\ f(x_*) \end{bmatrix} \sim N\left(\begin{matrix} u(x) \\ u(x_*) \end{matrix}, \begin{bmatrix} K + \sigma^2 I & k(x, x_*) \\ k(x_*, x) & k(x_*, x_*) \end{bmatrix} \right) \tag{S5}$$

where $K = \Sigma(x)$, and k is a variance function that is obtained from a kernel function (e.g., Gaussian kernel). Then, we can obtain

$$P(f(x_*) | Y, X, x_*) \sim N(u_*, \sigma_*^2) \tag{S6}$$

$$u_* = u(x_*) + k(x_*, x)(K + \sigma^2 I)^{-1}(Y - u(x)) \tag{S7}$$

$$\sigma_*^2 = k(x_*, x_*) - k(x_*, x)(K + \sigma^2 I)^{-1}(k(x, x_*) \tag{S8}$$

Through these methods, we can obtain conditional probability. Finally, we can identify the values of $f(x_*)$ and achieve predictions.

The Gaussian process regression is conducted through the Statistics and Machine Learning Toolbox MATLAB R2018a.

1.4 Computational cost

For the computational cost, we compare our FRMM with a recently popular and powerful recurrent neural network (e.g., reservoir computing). Our FRMM framework is integrated with three techniques, i.e., delay embedding, feature embedding, and Gaussian process regression. Delay embedding is used to construct a simple matrix from observed time series, whose computational cost is even low and can be negligible. For the feature embedding, our five algorithms both consist of three steps, where the first and second steps are common: the first step incorporates neighborhood information to construct a weighted graph and the third step is a spectral embedding step that involves an eigenequation computation. The second step is specific to the algorithm, transforming the neighborhood graph into suitable input for the spectral embedding step. These algorithms can be of two different kinds, either local methods (focus on the local geometry, e.g., LLE, Laplacian, Diffusion map, and LTSA) or global methods (focus on the global geometry, e.g., ISOMAP). Here, we first discuss the cost of individual representative algorithms, e.g., ISOMAP and LLE.

The ISOMAP algorithm consists of three steps: i) Finding the nearest neighbor for each data point based on the distance, the cost is approximately $O(N\log(K)L\log(L))$,

where K represents the number of nearest neighbor (we set $K = 8$ for all datasets in this work), N is the input dimension and L is the length of the series. ii) Computing the shortest path of graph based on Dijkstra's algorithm, which takes a cost $O(L^2(K+\log(L)))$. iii) Finding low-dimensional embedding via eigenvalue decomposition, it has a cost $O(EL^2)$. Thus, the cost of ISOMAP is approximately $O(N\log(K)L\log(L)) + O(L^2(K+\log(L))) + O(EL^2)$.

The LLE algorithm also has three steps: i) Searching nearest neighbors, the cost is approximately $O(N\log(K)L\log(L))$. ii) Constructing a weighted matrix, the cost is approximately $O(NLK^3)$. iii) Finding low-dimensional embedding via eigenvalue decomposition, it has a cost $O(EL^2)$. Then, the total cost of LLE is approximately $O(N\log(K)L\log(L)) + O(NLK^3) + O(EL^2)$.

The computational complexity of generic Gaussian process regression is approximately $O(N^3)$, where N represents the input dimension. In our framework, we set the E-dimensional feature manifold as the predictor, thus the complexity should be $O(E^3)$.

Thus, the total cost of FRMM:

$$C(FRMM(ISOMAP)) = O(N\log(K)L\log(L)) + O(L^2(K+\log(L))) + O(EL^2 + E^3) \quad \text{(S9)}$$

$$C(FRMM(LLE)) = O(N\log(K)L\log(L)) + O(NLK^3) + O(EL^2) + O(E^3) \quad \text{(S10)}$$

In a high-dimensional system, we often assume that $k \ll N, E \ll N$. Then, the cost can be approximated as

$$C(FRMM(ISOMAP)) = O(NL\log(L) + L^2(1+\log(L)) + L^2) \quad \text{(S11)}$$

$$C(FRMM(LLE)) = O(NL\log(L) + NL + L^2) \quad \text{(S12)}$$

When the length of the time series is larger than the number of observed variables, i.e., $L \geq N$, we have

$$\frac{L^2(1+\log(L))}{NL} > 1 \quad \text{(S13)}$$

Then, we infer

$$O(NL\log(L) + L^2(1+\log(L)) + L^2) > O(NL\log(L) + NL + L^2) \quad \text{(S14)}$$

$$TC(FRMM(ISOMAP)) > TC(FRMM(LLE)) \quad \text{(S15)}$$

Thus, the combination of ISOMAP has a higher computational cost than the combination of LLE. ISOMAP is a global approach, which searches nearest neighbors between all the pairs. It would take a higher cost compared with other local approaches.

Reservoir computing is a powerful recurrent neural network for the predictions in complex systems, especially chaotic systems. A general reservoir computing scheme consists of an input layer, a reservoir network, and an output layer [1]. When predicting T-step forward, RC first maps an N-dimensional input data to a D-dimensional vector by the $D \times N$ matrix. Then, RC conducts predictions from the reservoir to the output module by the $T \times D$ matrix. There may need M neurons in the reservoir network, and the time cost of the processing of reservoir converting is $O(D^3)$. Suppose there are S iterations from reservoir to output, the time cost for the transformation is approximately $O(2SN(2/3T^3 + 2T^2))$. The total cost of generic RC is approximately $O(2SN(2/3T^3 + 2T^2) + D^3)$.

In general, it is difficult to conduct accurate even longer predictions in chaotic systems. Thus, T is a relatively small value. For a generic RC, the reservoir has a much higher dimension [1], i.e., $N \ll D$. Thus, the main computational cost for a generic RC can be determined as $O(D^3)$.

According to Eq. (S11), when $L < N \ll M$, we obtain

$$O(NL\log(L) + L^2(1+\log(L)) + L^2) < O(L^2(N+2+\log(L)) < O(N^2(N+2+N)) \quad \text{(S16)}$$

$$O(N^2(N+2+N)) \approx O(N^3 + N^2) < O(D^3) \quad \text{(S17)}$$

Generally, FRMM has a lower computational cost than the generic reservoir computing network.

1.5 Remark on real-world datasets

Our FRMM framework is designed for complex dynamical systems. Model systems are often depicted by some differential equations and system components are known and networked in certain rules. However, one needs to select a subsystem as well as its components for real-world datasets. In dynamical systems theory, causally linked variables share a common attractor (Ref. 28 in main text). If a variable is completely irrelated to other variables in a given subsystem, it is risky to add it to this subsystem. This irrelated variable does not share a common attractor with other variables and it does not belong to the given dynamical system (e.g., the EEG signals from two unrelated people may belong to individual dynamical systems, and their EEG signals can not be considered in a common subsystem in our work), thus its time series

may not be used to reconstruct an isomorphic attractor with the given subsystem by delay embedding. Though identifying causal interactions for real-world samples is still an open issue across many disciplines, it is better to select a real-world subsystem, whose components are possibly and intuitively networked.

1.6 Supplemental remark on FRMM framework

The FRMM framework is an innovative combination of delay embedding and feature embedding. It is needed to discuss the necessity of that combination. Here, we first discuss the theoretical foundation of separate approaches, including the framework of delay embedding and Gaussian process regression, and the framework of feature embedding and Gaussian process regression. Then, we conduct experiments to make comparisons of separate approaches with FRMM.

If we use feature embedding alone (including Gaussian process regression), the prediction task is operated as many generic regression approaches, which first identify optimal features as predictors by feature embedding. Then, one can conduct predictions by training correlations between input features and target variables. For example, $f(Y_t) = y_{t+T}$, Y consists of the optimal features (series) from the original system, y is a target variable, T is the predicted step. This framework often outputs multi-step-ahead predictions indirectly, e.g., iteration (predicted values are used as input to find longer predictions), which means that we need to conduct T experiments for T step predictions, where accumulated errors may lead to poor performance with the increase of predicted horizons. Also, multiple training processes often need relatively higher time cost. However, FRMM enables us to conduct multi-step-ahead predictions directly through the combination of delay embedding.

If we use delay embedding alone (including Gaussian process regression), which has been discussed in work (cf. 44 in main text). From the combination of delay embedding and Gaussian process regression, one can construct a prediction framework that builds a mapping from the original system to the reconstructed manifolds, see Eq. (S18).

$$
\psi_i \begin{pmatrix} x_1(1) & x_2(1) & \cdots & x_N(1) \\ \vdots & \vdots & \cdots & \vdots \\ x_1(h) & x_2(h) & \cdots & x_N(h) \\ \vdots & \vdots & \cdots & \vdots \\ x_1(L) & x_2(L) & \cdots & x_N(L) \end{pmatrix} = \begin{pmatrix} x_i(1) & x_i(1+\tau) & \cdots x_i(1+(E-1)\tau) \\ \vdots & \vdots & \cdots & \vdots \\ x_i(h) & x_i(h+\tau) & \cdots & x_i(L) \\ \vdots & \vdots & \cdots & \vdots \\ x_i(L) & x_i(L+\tau) & \cdots x_i(L+(E-1)\tau) \end{pmatrix} \quad \text{(S18)}
$$

where the left matrix represents the points in the original system and the right matrix is identified by delay embedding from a target time series. $h = L-(E-1)\tau$ and L is the length of the time series. This framework has been certified reliable in various systems with several components. However, it shows limitations for some high-dimensional systems since it is not always guaranteed to conduct an isomorphic topology with an even larger embedding dimension. Moreover, high-dimensional real-world systems often show redundant information that may negatively affect the predictions. We apply the 90-dimensional Lorenz system as an example, the combination of delay embedding and Gaussian process regression (single Gaussian process regression) exhibits poor performance, see Fig. S13).

In summary, the integration of delay embedding and feature embedding overcomes the curse of dimensionality and helps to directly achieve accurate predictions for all components in complex systems.

1.7 Supplemental remark on feature embedding

In this work, we use classic manifold learning algorithm to find faithful low-dimensional representation of a high-dimensional system. A fundamental basis of these algorithms is to preserve geometry features of the original system in a low-dimensional space, e.g., the nearest neighbors in the original system are also nearest neighbors in its low-dimensional representation. However, real-world systems may show different topological structures and then exhibit quite different geometry features, there is no well-accepted algorithm that is faithful for all the systems. On this basis, several advanced feature embedding algorithms are proposed for different geometries or similar geometries but with different aspects, e.g., ISOMAP, LLE, Laplacian, Diffusion map, and LTSA [2]. ISOMAP finds low-dimensional representation based on global geodesic distance (different from Euclidean metric) and is available for the manifold as a convex region distorted in certain ways, such as folding or twisting. Unlike ISOMAP, LLE, Laplacian, Diffusion map, and LTSA focus on local geometry. LLE assumes that

data on a manifold can be approximated by linear combinations of their nearest neighbors, it performs well when the data are uniformly sampled over the manifold but shows limitations for some closed manifolds, e.g., sphere and torus. Laplacian algorithm is closely related to LLE, which measures the local feature by an isotropic diffusion kernel. Diffusion map approximates local geometry by diffusion distance. LTSA utilizes the tangent space in the neighborhood of a data point to represent the local geometry.

Still, it is challenging to give reliable criteria for the selection of algorithm without enough prior knowledge of system topological structure [2], especially in real-world systems, only from observed time series, it is not easy to view their geometries in a high-dimensional space. Nevertheless, the selected techniques make sense for many systems. In practical applications, it is possible to compare them and find an optimal one, since these algorithms are computationally efficient that they often use eigenvalue decomposition without complex iterative processes. Take the 90-dimensional Lorenz system as an example, we calculate the time costs for different feature embedding algorithms. All the algorithms show low computational costs, see Table. S3.

1.8 Parameter selection

Both embedding dimension (E) and time delay (τ) are important parameters to conduct delay embedding. Theoretically, an isomorphic reconstruction is guaranteed on the condition $E > 2d$, where d represents the box dimension of the attractor [3]. The estimated box dimensions in selected systems are provided in *SI Appendix* Table S4. In this work, we use the false nearest neighbor (FNN) and mutual information to determine the embedding dimension and time lag, respectively. In practice, the selection of time lag is flexible that several different lags can be used to find available reconstruction, see *SI Appendix* Fig. S9. Note that the predicted step is determined by $T = (E-1)\tau$, we can test the predicted horizons by increasing the time lags. However, it is still challenging to achieve reliable even longer predictions in complex systems, since the reconstructed attractor may exhibit a completely different topology from the original system when applying a large time lag.

1.9 Remark on predictor

Our FRMM framework sets low-dimensional feature manifold as a generalized predictor for the prediction of all components in a complex system. Several representative model and real-world systems are applied to show the efficiency of our approach. To further illustrate its effectiveness, we verify on two model systems including a chaotic system and generic nonlinear system, e.g., the 3-dimensional Chen chaotic system (Eq. (S19)) and a 3-dimensional ecology system (Eq. (S20) (non-chaotic dynamics), and two real-world systems from physiological system and climate system, i.e., the series from a physiological system (The sample datasets are freely downloaded at https://www.physionet.org/physiobank/database/santa-fe/) and the index and sea surface temperature in ENSO cycle (The datasets are free on https://www.cpc.ncep.noaa.gov). By setting the feature manifold as a generalized predictor, FRMM achieves reliable predictions of all components in these systems, see Fig. S14 and S15.

$$\begin{cases} \dot{x} = c_1(y-x), \\ \dot{y} = c_2 x(1+z) + c_3 y, \\ \dot{z} = xy - c_4 z, \end{cases} \tag{S19}$$

where $c_1 = 35, c_2 = -7, c_3 = 28$. With an initial state $(x(0), y(0), z(0)) = (1,2,3)$ and integrated interval [0,29], we obtain three time series. Each of them has a length of 1557. FRMM yields accurate predictions for all the components (Fig. S14(a)).

$$\begin{aligned} x(t+1) &= x(t)[4 - 4x(t) - 2y(t) - 0.4z(t)], \\ y(t+1) &= y(t)[3.1 - 0.31x(t) - 3.1y(t) - 0.93z(t)], \\ z(t+1) &= z(t)[2.12 + 0.636x(t) + 0.636y(t) - 2.12z(t)], \end{aligned} \tag{S20}$$

From the initial state $(x(0), y(0), z(0)) = (0.4, 0.5, 0.6)$ and time interval $t = 1, \cdots, 399$, the output times series has a length of 400. By setting the feature manifold as the predictor, we achieve accurate ahead predictions for all variables, see Fig. S14 (b).

To further certify the universality of our predictor (feature manifold), we explain it with mathematical processes. Given an *N*-dimensional dynamical system with original manifold *M*, FRMM aims to find its low-dimensional representations from different approaches.

According to the manifold learning technique (feature embedding), for each state point on M, we can find a corresponding state point in a low-dimensional representation M_0 ($M_0 \subseteq R^E, E \ll N$) via a mapping ϕ , and this mapping exists and homeomorphism [4]. This process can be given mathematically, $\forall X(t) \in M$, $Y(t) \in M_0$, we have (S21)

$$Y(t) = \phi(X(t)), t = 1, 2, \cdots, L. \tag{S21}$$

Since ϕ is a homeomorphism, we deduce an inverse form (S22)

$$X(t) = \phi^{-1}(Y(t)). \tag{S22}$$

On the other hand, in a dynamical system, each time series variable can be utilized to reconstruct an isomorphic manifold M_{x_i} ($M_0 \subseteq R^E, E \ll N$) based on delay embedding theory. This process can be also given mathematically, $\forall X(t) \in M$, $\tilde{X}_i(t) \in M_{x_i}$, we have (S23)

$$\tilde{X}_i(t) = \varphi_i(X(t)), t = 1, 2, \cdots, L, i = 1, 2, \cdots, N. \tag{S23}$$

Considering Eq. (S22) and Eq. (S23), we obtain (S24)

$$\tilde{X}(t) = \varphi_i(\phi^{-1}(Y(t))). \tag{S24}$$

Thus, $\forall \tilde{X}_i(t) \in M_{x_i}$, $Y(t) \in M_0$, we have (S25)

$$\psi_i(Y(t)) = \tilde{X}_i(t), \ \ i = 1, 2, \cdots, N, \tag{S25}$$

where $\psi_i(x) = \varphi_i \phi^{-1}(x)$.

Consequently, we obtain (S26)

$$\psi_1(Y(t)) = \tilde{X}_1(t), \cdots, \psi_N(Y(t)) = \tilde{X}_N(t), \tag{S26}$$

where $Y(t) \in M_0$ and $\tilde{X}_i(t) \in M_{x_i}$.

From Eq. (S26), the feature manifold M_0 is used as a generalized and fixed predictor and is available to predict future dynamics for all components in complex systems.

2 Supplemental Figures

2.1 Low-dimensional embeddings from noisy Lorenz and Rössler systems

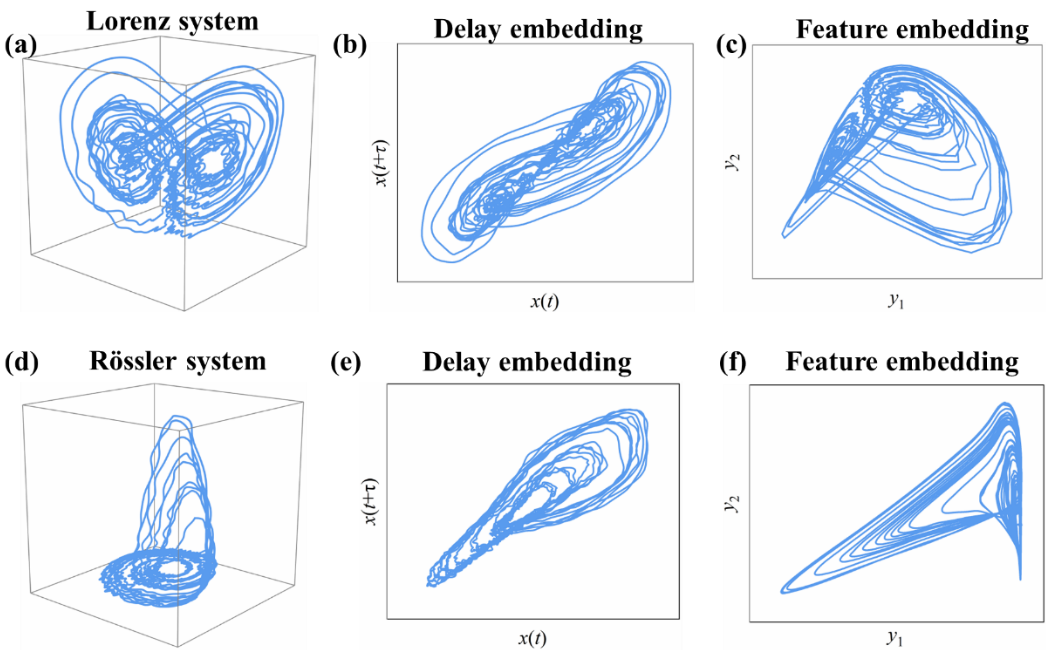

Fig. S1 Low-dimensional embeddings from noisy Lorenz and Rössler systems. Based on delay embedding and feature embedding, one can find low-dimensional representations of the Lorenz system (a-c) and Rössler system (d-f) with additive noise ($\sigma = 0.5$, σ represents noise strength).

2.2 The prediction of the Rössler system

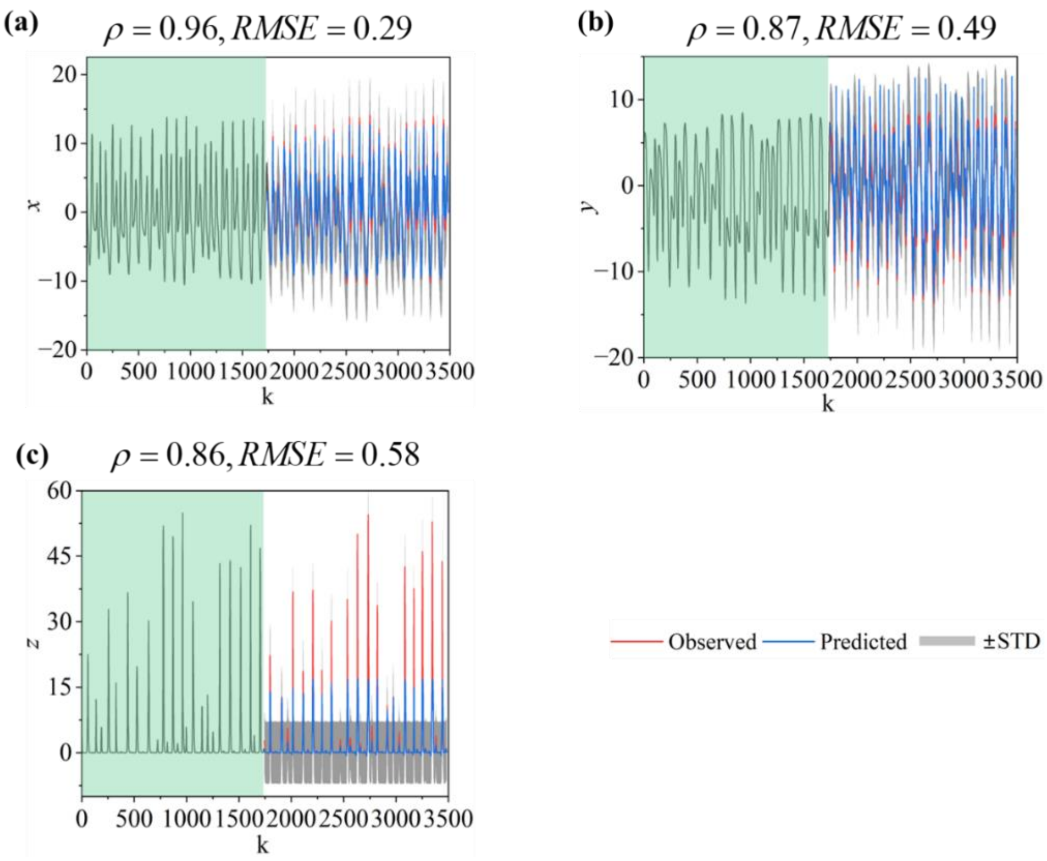

Fig. S2 The prediction of the Rössler system. Based on our FRMM, we embed the 3-dimensional Rössler system into 2-dimensional space via the LTSA algorithm and delay embedding (see Fig. 1 in main manuscript). FRMM achieves 8-step-ahead ($E = 2, \tau = 10$) predictions for all the components (a-c). We validate the accuracy by randomly selecting 50% of the observed data as training samples (green shaded area), and the others are test samples. The average correlation between the predicted values and observed values is 0.9, and the average error is $RMSE = 0.45$.

2.3 The prediction of daily and monthly wind speeds

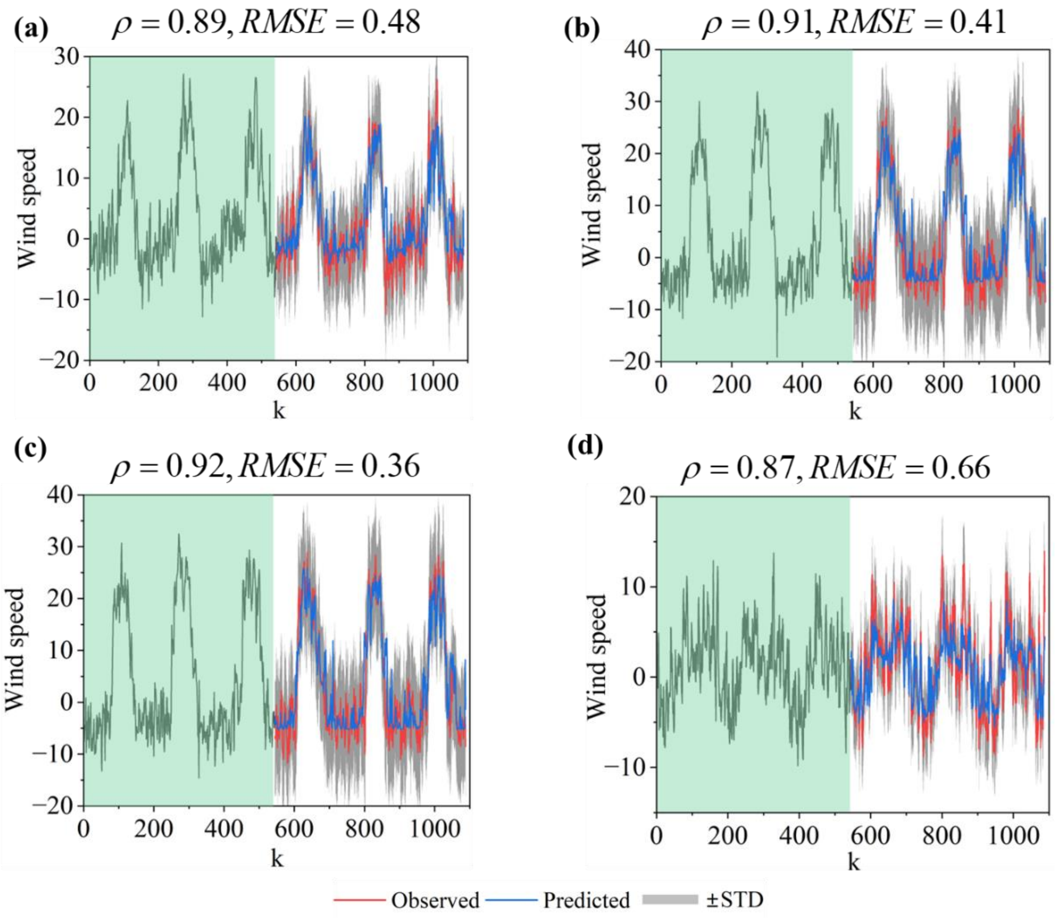

Fig. S3 The predictions of daily wind speed. We randomly show the performances of four series, as supplementary to Figs. 4(a-c) in the main manuscript. FRMM can achieve multistep ahead predictions for daily wind speeds.

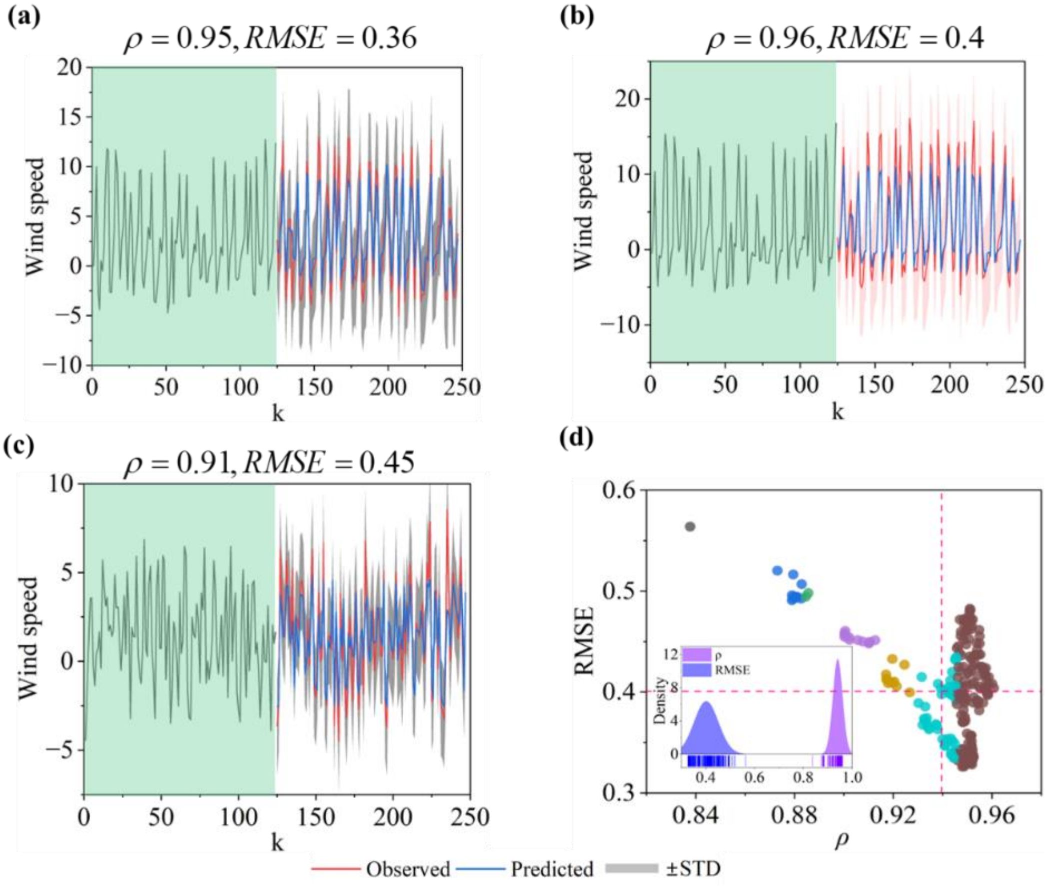

Fig. S4 The predictions of monthly wind speed. We first randomly show the performances of three series (a-c). Then, we give the performances for all series by the distributions of metrics ρ and RMSE (d). FRMM achieves 20-step ($E = 5, \tau = 5$) ahead predictions for all the components. The average correlation between the predicted values and observed values is $\rho = 0.94$, and the average error is $RMSE = 0.41$.

2.4 The predictions of the EEG signals

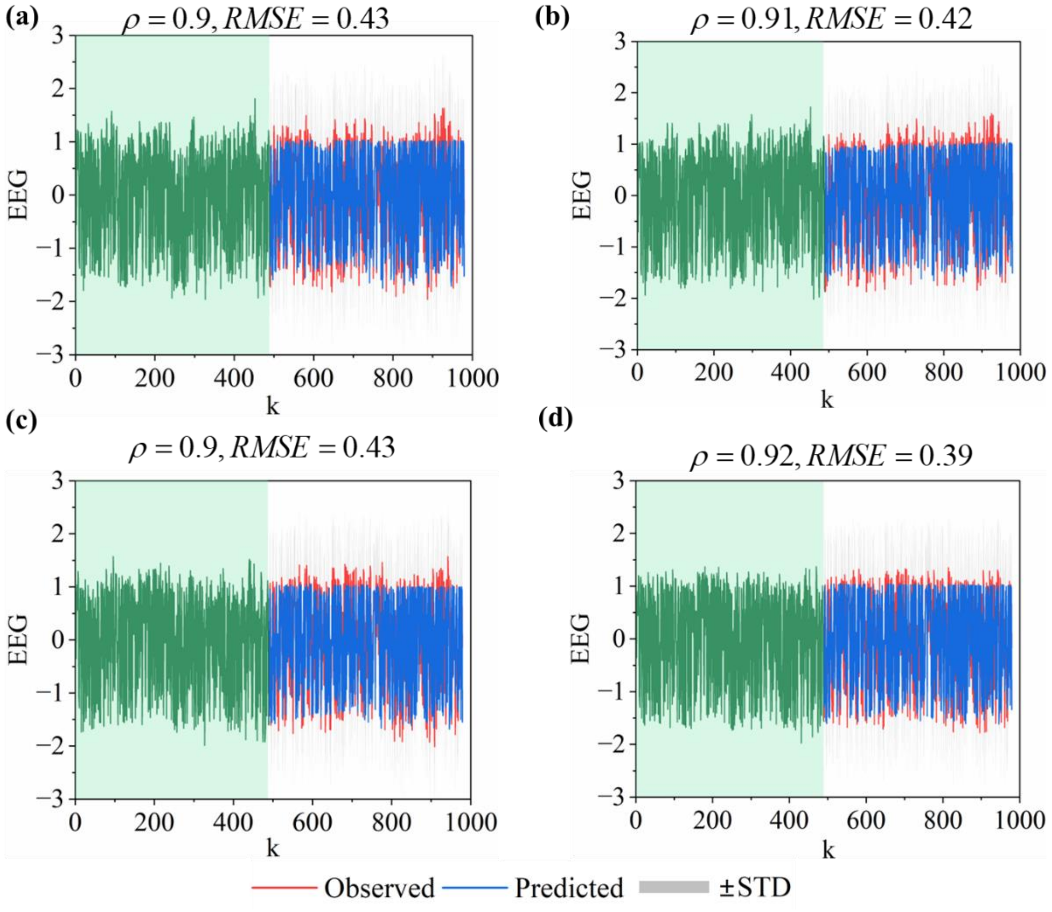

Fig. S5 The predictions of EEG signals. We randomly show the performances of four series, as the supplementary of Figs. 4(d-f) in the main manuscript. FRMM can achieve multistep ahead predictions for EEG signal series.

2.5 The predictions of exchange rates

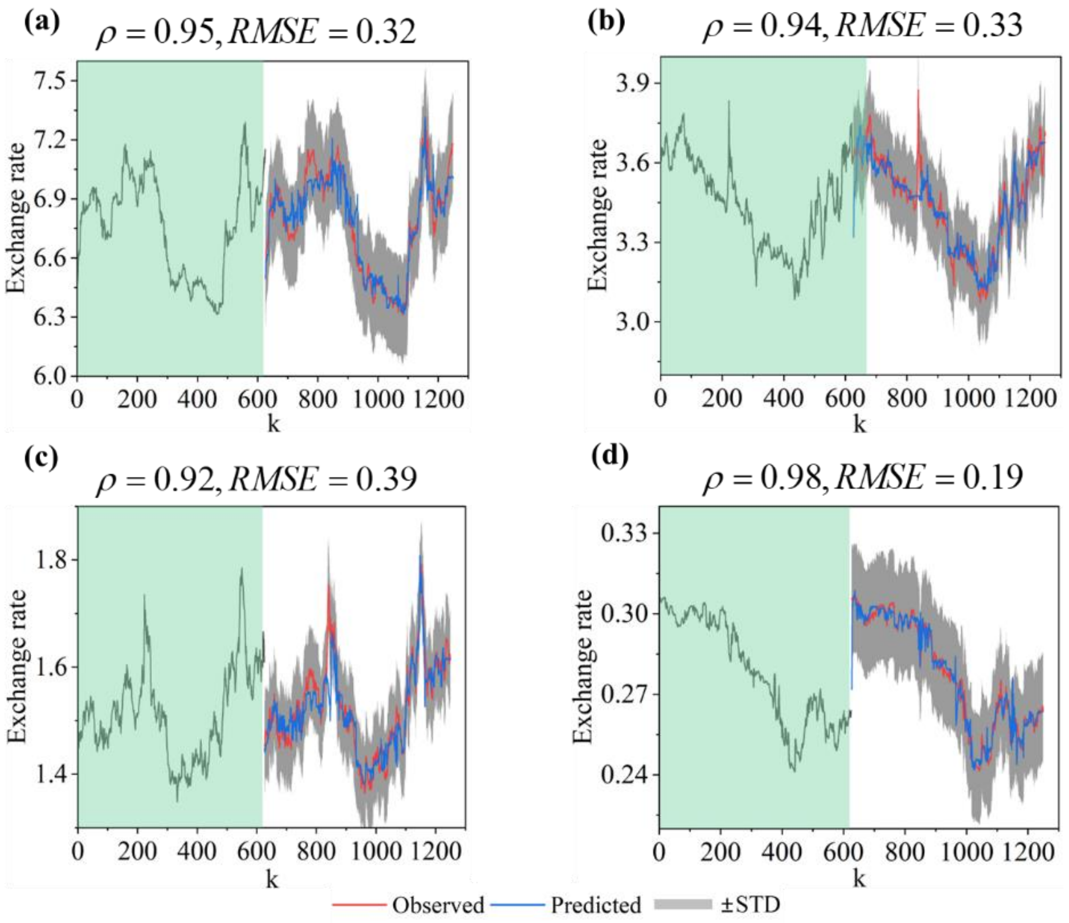

Fig. S6 The predictions of exchange rates. We randomly show the performances of four price series, as the supplementary of Figs. 4(g-i) in the main manuscript. FRMM can achieve multistep ahead predictions for exchange rates.

2.6 The predictions of the stock index

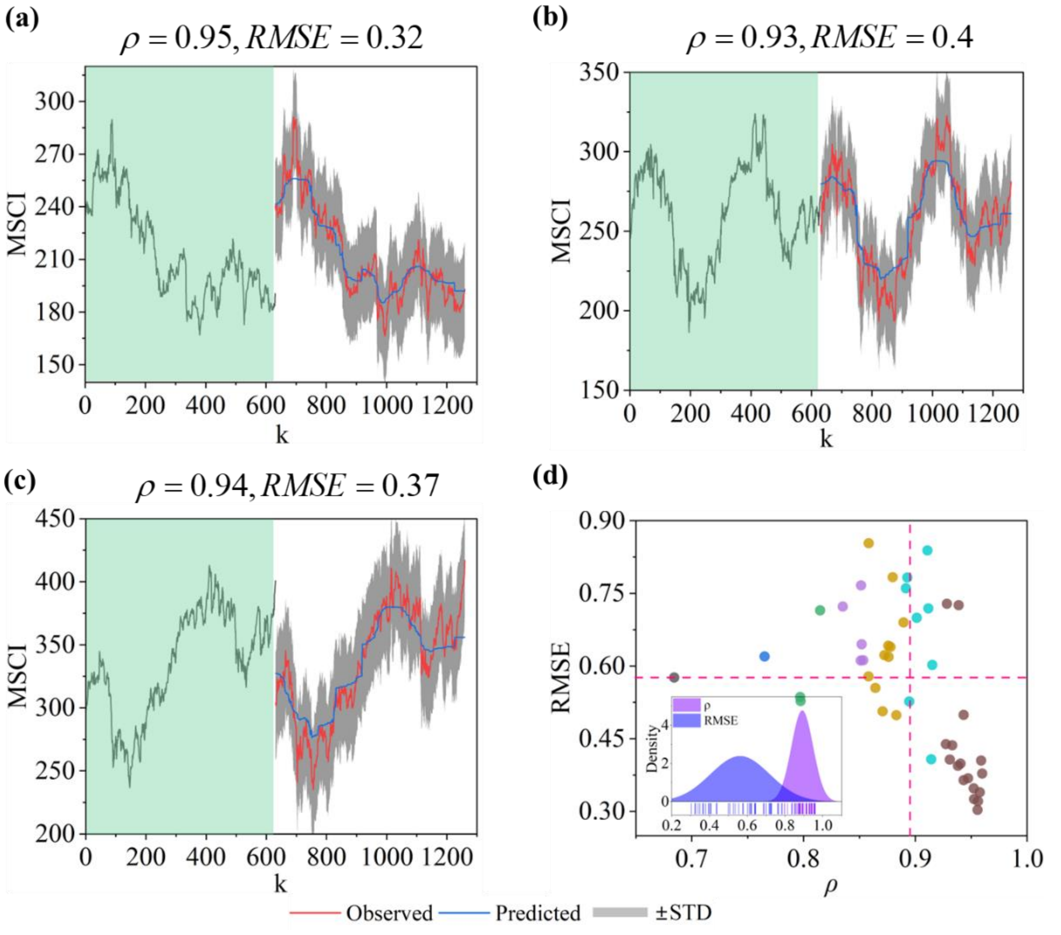

Fig. S7 The predictions of the stock Morgan Stanley Capital International index (MSCI). We first randomly show the performances of three series (a-c). Then, we give the performance for all series by the distributions of metrics ρ and RMSE (d). FRMM yields accurate 20-day-ahead predictions for all observations, where the average ρ and RMSE are 0.88 and 0.57, respectively.

2.7 The predictions of traffic speeds

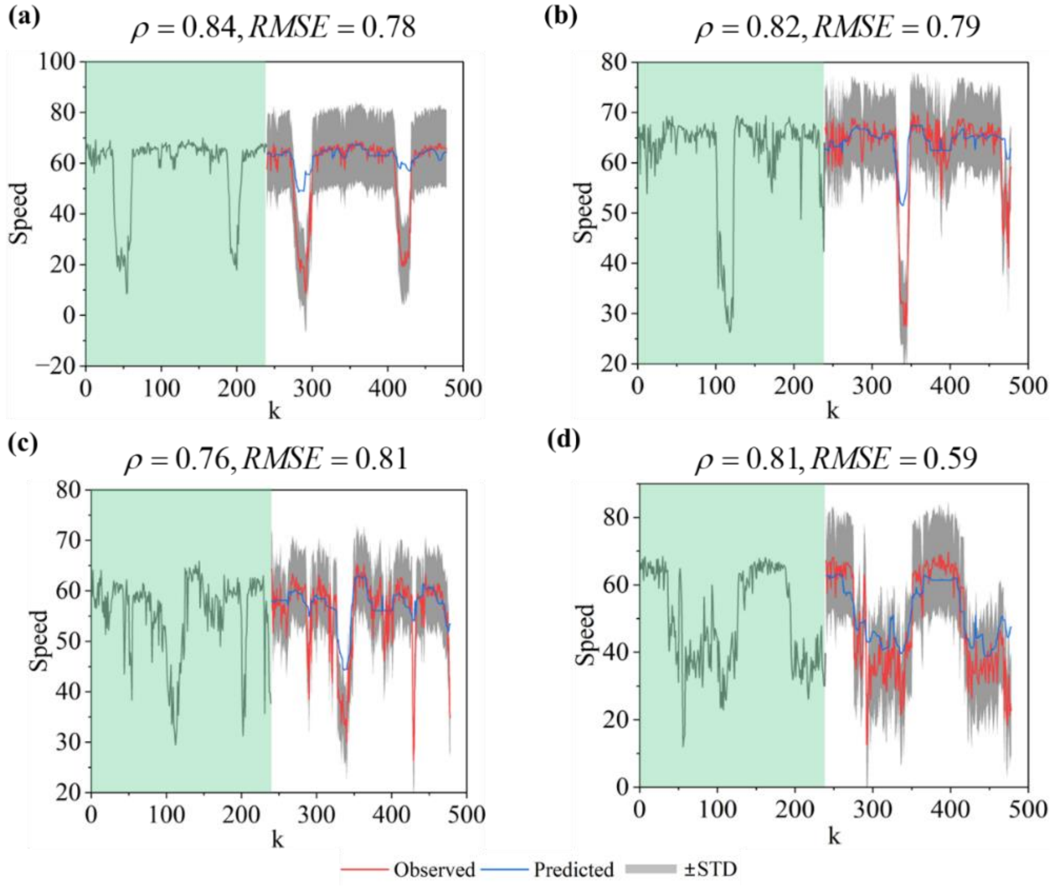

Fig. S8 The predictions of traffic speeds. We randomly show the performances of four price series, as the supplementary of Figs. 4 (j-l) in the main manuscript. FRMM framework can conduct multistep ahead predictions for traffic speeds.

2.8 The reconstructed manifold with different lags τ

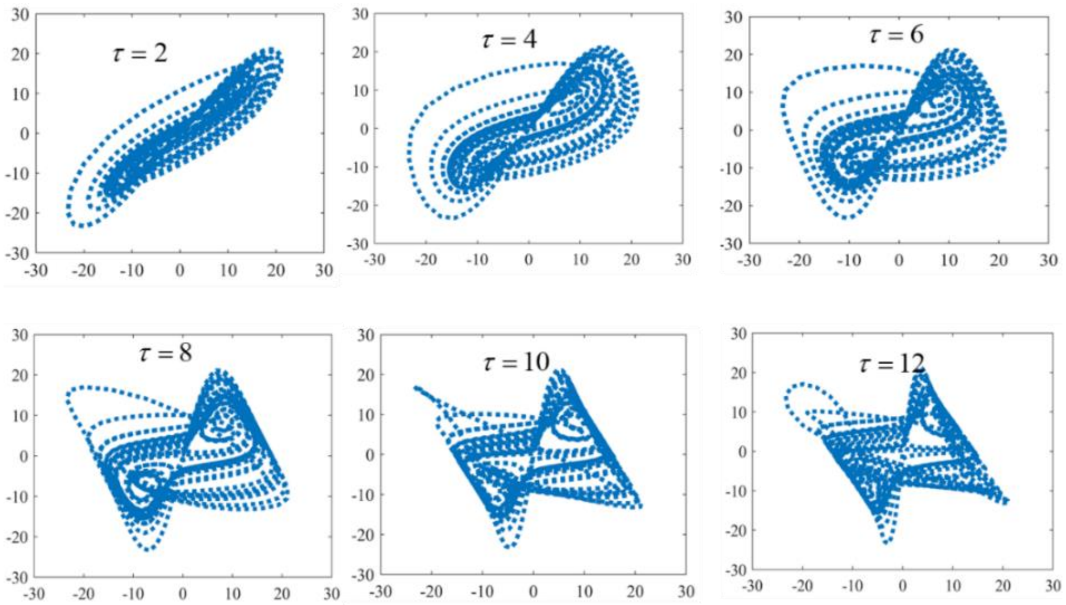

Fig. S9 The reconstructed manifolds with different lags (τ). The results show that one can reconstruct 2-dimensional attractors of the 3-dimensional Lorenz system through different time lags τ . Therefore, it is possible to operate relatively longer step predictions by increasing the lag (predicted step $T=(E-1)\tau$). However, the reconstructed attractor is not isomorphic with the original attractor with a much larger time lag. This gives the limitation of our framework to achieve longer-term predictions in complex systems.

2.9 The length of the training sample and test sample

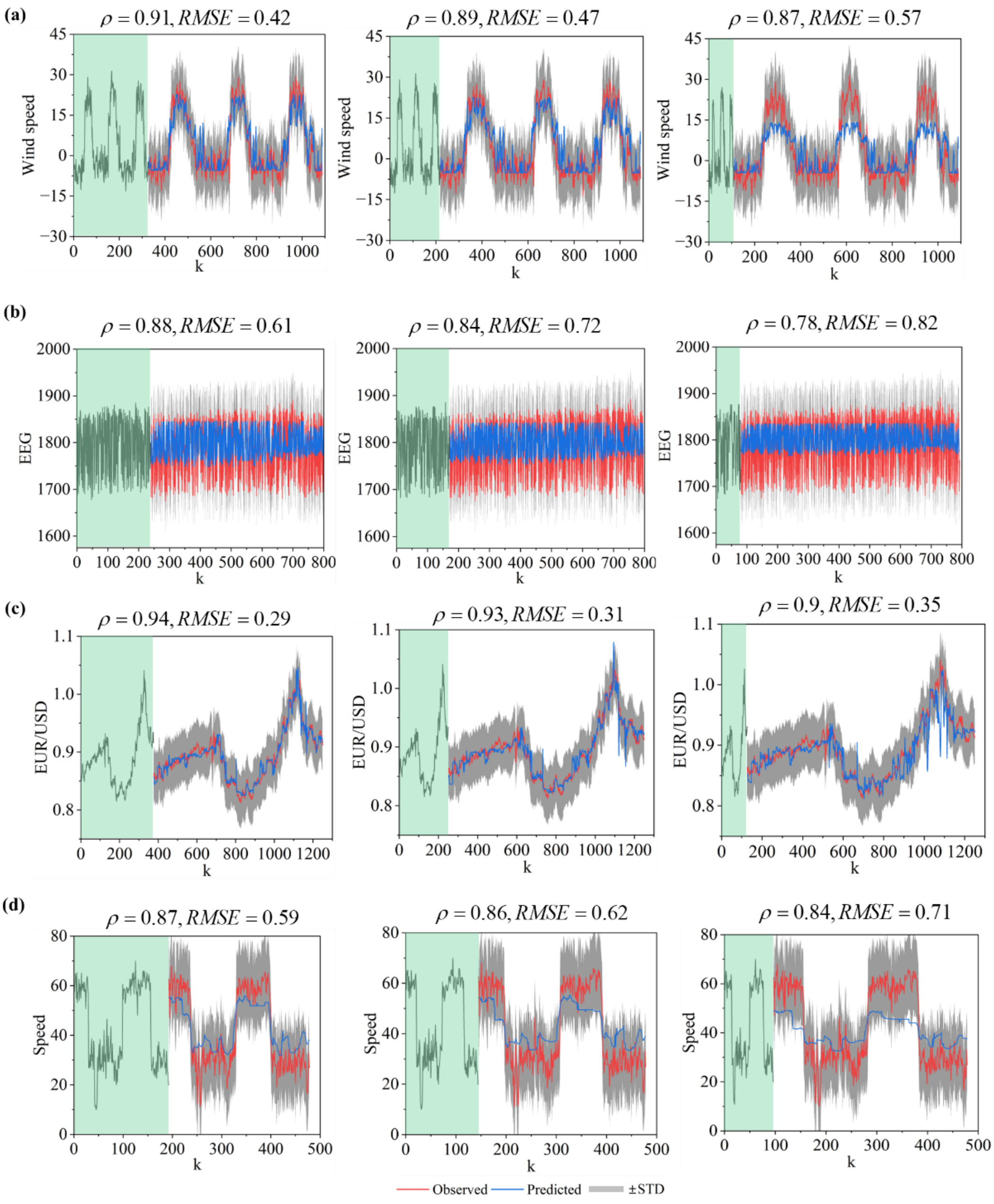

Fig. S10 Robustness test on the length of training samples in real-world systems. In this work, we validate the performance by randomly selecting a part of the observed series as a training sample, and others are test samples. Here, we evaluate our framework by setting the proportions of the training sample to 30% (first column), 20% (second column), and 10% (final column). Our framework is reliable even with a short training sample (e.g., 10%). (a) The performance of wind speed. (b) The performance of the EEG signal. (c) The performance of exchange rate. (d) The performance of traffic speed.

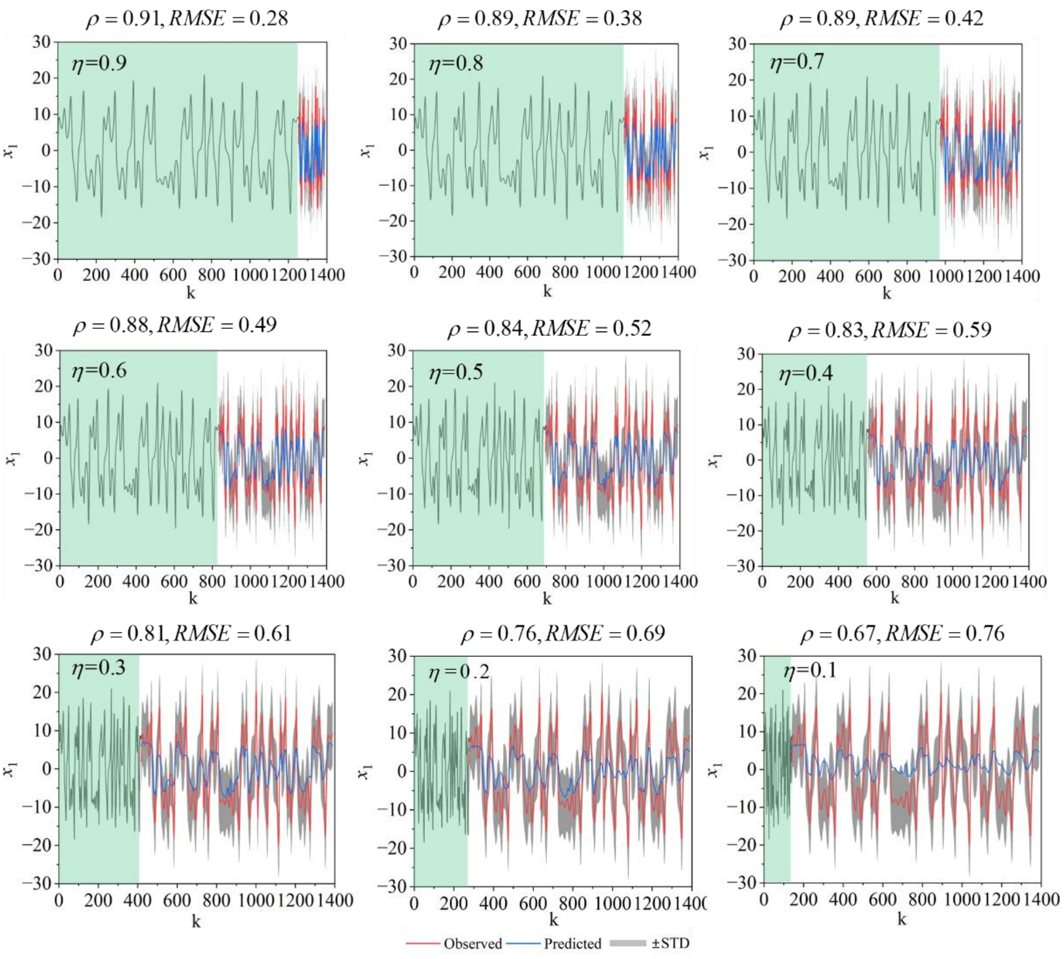

Fig. S11 Robustness test on the length of the training sample. Our framework is robust with short and longer training series. η ($0.1 \le \eta \le 0.9$) represents the proportion of the training sample. The 90-dimensional ordinary Lorenz system is used as an example.

2.10 Robustness tests

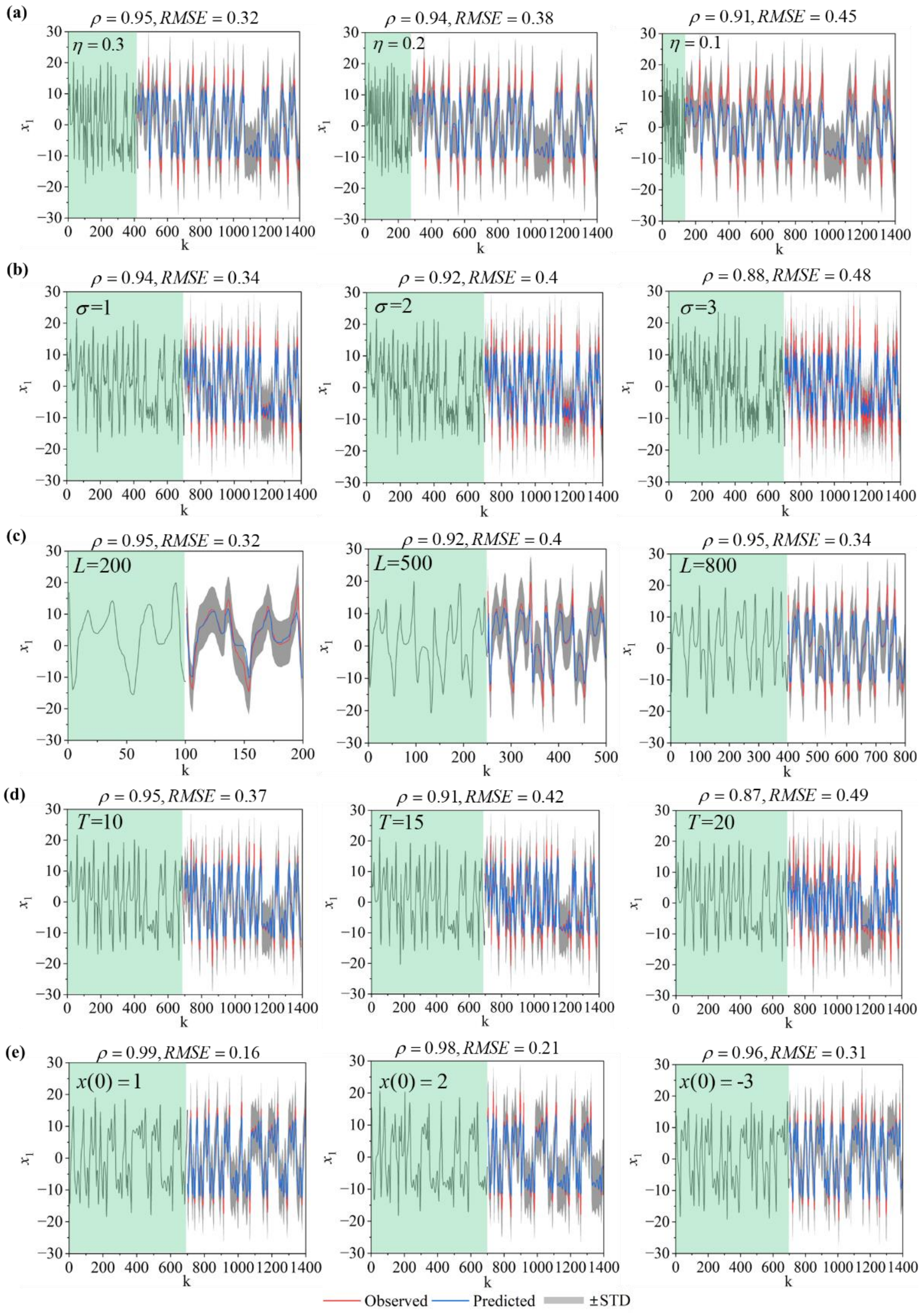

Fig. S12 The robustness tests include the length of the training sample (a), additive noise (b), length of the observed series (c), predicted step (d), and initial state (e). The 3-dimensional Lorenz system is used as an example.

2.11 The prediction by separate approaches from FRMM

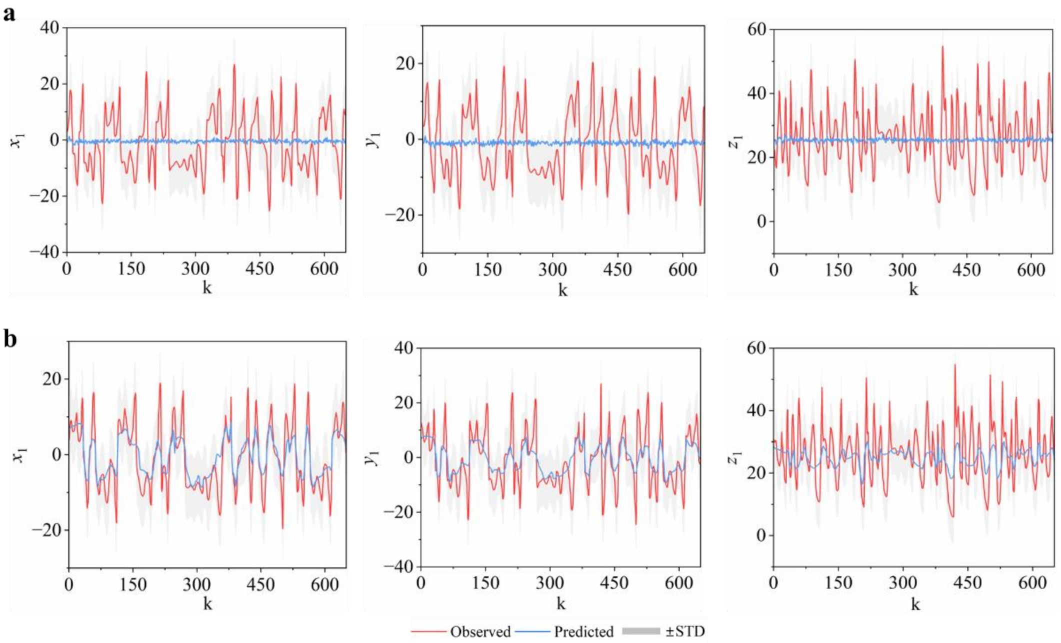

Fig. S13 The performance of 90-dimensional ordinary Lorenz system. (a) We use a predictive model with a combination of delay embedding and single task Gaussian process regression, where all the variables are used as predictors ($E=11, \tau=1$). (b) The prediction results via a combinative model from feature embedding and Gaussian process regression. We first select 11 ($E=11$) features of the Lorenz system via diffusion map, and then we operate predictions by training correlations between these features and a target variable. Results show that neither feature embedding nor delay embedding framework can be separately used for reliable predictions of all components in complex systems, while the integration of delay embedding and feature embedding (FRMM) has the potential to address that prediction task (see Fig. 3 (d-f) in main text).

2.12 The prediction of other model and real-world systems

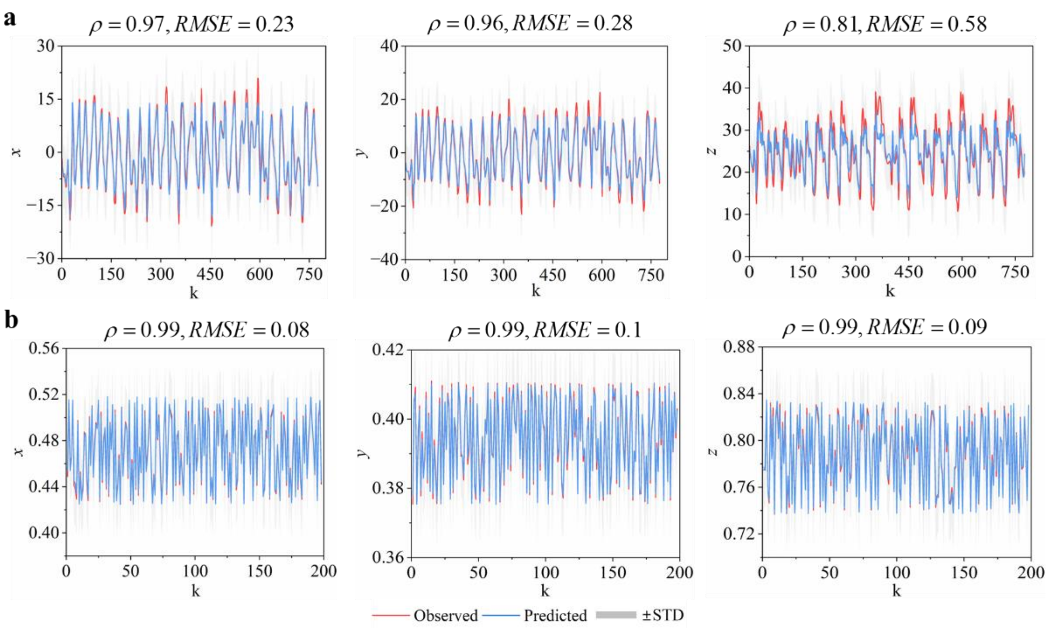

Fig. S14 The performance in the 3-dimensional Chen system (a) and an ecology system (b). By setting the feature manifold as the predictor, we achieve accurate predictions for all components in complex systems. Note: we validate FRMM by randomly selecting 50% of the series as a test sample. Diffusion map is utilized to find the feature manifold, and $E = 2, \tau = 8$.

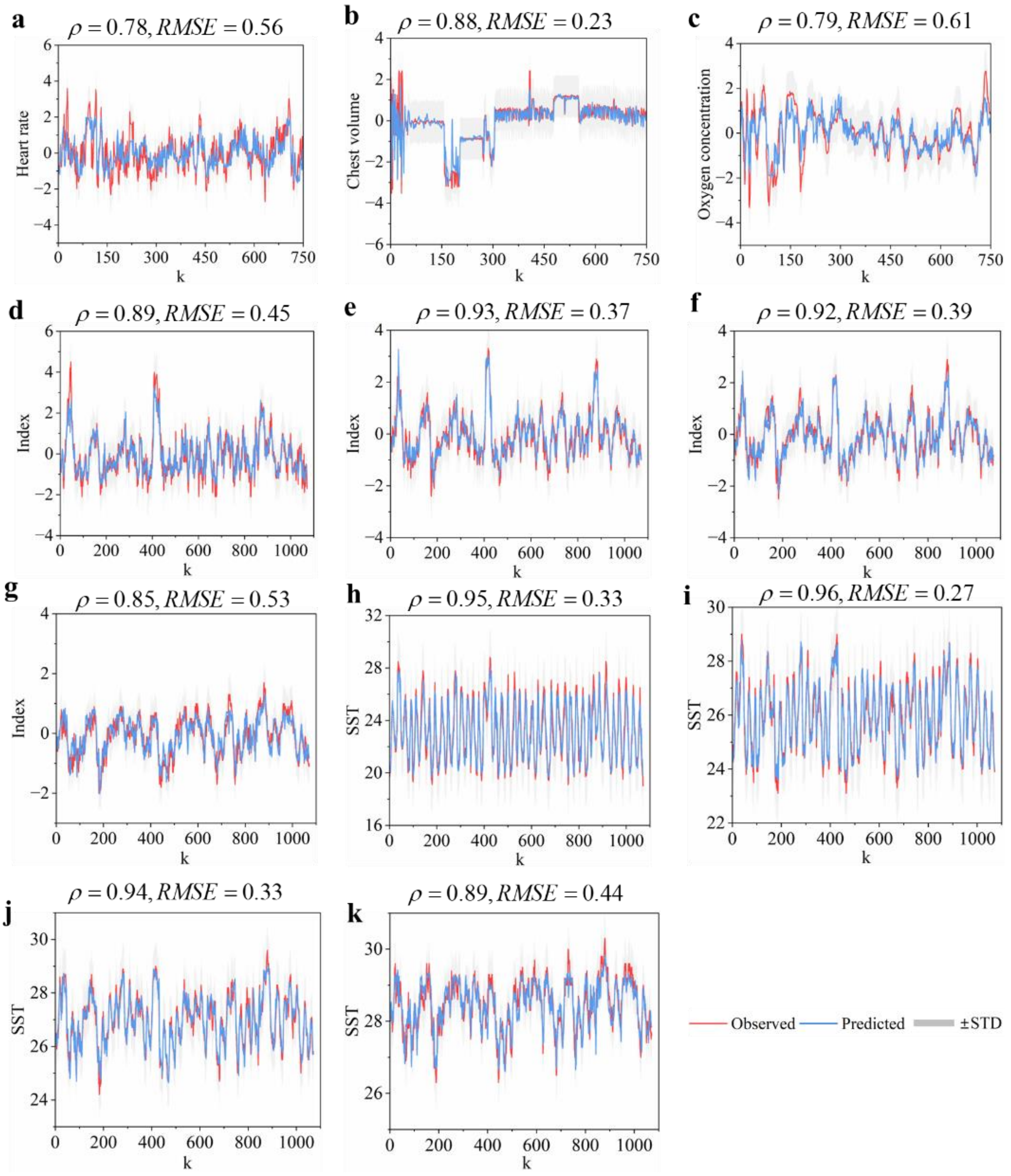

Fig. S15 The performance in heart-lungs-blood oxygen concentration system (a-c) and ENSO cycle (d-k). By setting their feature manifolds as generalized predictors, we make predictions for all components in these systems. Note: Heart-lungs-blood oxygen concentration system contains three time series variables, i.e., heart rate, chest volume, and blood oxygen concentration. Due to different units, we normalize all the series against their mean and standard deviation. The ENSO cycle consists of four anomaly series (index) and four sea surface temperature (SST) series in ENSO 1+2, ENSO 3, ENSO 3.4, and ENSO 4 regions. *k* represents the randomly selected test sample (50% of the entire series).

3 Supplemental Table

3.1 The sample of exchange rate

Table S1 Selected exchange rates in this work

EUR	SGD	AUD	EEK	MAD	CLP
GBP	HKD	NZD	FIM	PEN	CYP
CHF	INR	CND	GRD	PTE	EGP
SEK	TWD	BHD	HNL	RON	FJD
NOK	MXN	BGN	HUF	SKK	XAF
TRY	BRL	HRK	ISK	SIT	XPF
RUB	ARS	CZK	JMD	ESP	GHS
JPY	ZAR	DKK	KWD	UYU	HNL
RSD	SAR	NLG	LUF	DZD	IDR
CNY	ILS	XCD	MTL	BEF	IEP
ITL	MYR	PLN	RSD	LKR	THB
TDD	AED	VND	ZMW		

Note: US dollar (USD) is numeraire.

3.2 The sample of stock index

Table S2 The 46-dimensional stock index system

CHN	FRA	NZL	USA	THA	TW
AUS	DEU	NOR	SGP	IRS	COL
AUT	HK	PRT	RUS	MEX	CZE
CAN	IRL	SWE	EGY	PHL	GRC
DNK	ITA	CHE	IDN	BRA	HUN
FIN	JPN	GBR	MYS	CHI	IND
ARG	LKA	JOR	PAK	TUR	MAR
KOR	PER	POL	ZAF		

Note: HK represents Hong Kong, China. TW represents Tai Wan, China.

3.3 An example of time cost for feature embedding

Table S3 Time costs of different feature embedding algorithms

Algorithm	Time cost (Second)
ISOMAP	12.3919
LLE	0.32629
Laplacian	0.14581
Diffusion map	0.29983
LTSA	0.21549

Note: we identify the low-dimensional ($E = 11$) representation of the 90-dimensional Lorenz system via different algorithms. All algorithms show relatively low time costs.

3.4 The estimated box dimension in our datasets

Table S4 The estimated box dimension in selected systems

Objection	Embedding dimension (E)	Box dimension (d)	Feature embedding
The 3-dimensional Lorenz system	2	0.91	Diffusion map
The 3-dimensional Rössler system	2	0.92	LTSA
The 90-dimensional ordinary Lorenz system	11	1.51	Diffusion map
The 90-dimensional Lorenz system with varying dynamics	11	1.59	Diffusion map

The 231-dimensional wind speed system	5	1.68	LLE
The 64-dimensional EEG signal system	5	1.74	LTSA
The 70-dimensional exchange rate system	5	1.36	ISOMAP
The 46-dimensional stock price system	5	1.33	Laplacian
The 207-traffic speed system	10	1.98	Laplacian

Note: The box dimension is estimated by the FRACLAB Toolbox in MATLAB R2018a.

4 Supplemental References

1. Jiang, J. J. & Lai, Y. C. Model-free prediction of spatiotemporal dynamical systems with recurrent neural networks: Role of network spectral radius. *Physical Review Research*. 1, 033056 (2019).
2. Ma, Y. & Fu, Y. Manifold learning theory and applications (1st ed.). CRC Press. https://doi.org/10.1201/b11431 (2011).
3. Kraemer, K. H. et al. A unified and automated approach to attractor reconstruction. *New Journal of Physics*. 23, 033017 (2021).
4. Coifman, R. R. & Lafon, S. Diffusion maps. *Applied and Computational Harmonic Analysis*. 21, 5-30 (2006).
